# Supplementary material for: The insulin/IGF signaling cascade modulates SUMOylation to regulate aging and proteostasis in Caenorhabditis elegans
Source: eLife. 2018 Nov 7;7:e38635. doi: 10.7554/eLife.38635 (PMC6277199; doi:10.7554/eLife.38635)
Supplement: Transparent reporting form [file elife-38635-transrepform.pdf]

1st Floor
24 Hills Road
Cambridge CB2 1JP, UK

P 01223 855340
W elifesciences.org
T @elife

eLife's transparent reporting form

We encourage authors to provide detailed information *within their submission* to facilitate the interpretation and replication of experiments. Authors can upload supporting documentation to indicate the use of appropriate reporting guidelines for health-related research (see EQUATOR Network), life science research (see the BioSharing Information Resource), or the ARRIVE guidelines for reporting work involving animal research. Where applicable, authors should refer to any relevant reporting standards documents in this form.

If you have any questions, please consult our Journal Policies and/or contact us: editorial@elifesciences.org.

Sample-size estimation

- You should state whether an appropriate sample size was computed when the study was being designed
- You should state the statistical method of sample size computation and any required assumptions
- If no explicit power analysis was used, you should describe how you decided what sample (replicate) size (number) to use

Please outline where this information can be found within the submission (e.g., sections or figure legends), or explain why this information doesn't apply to your submission:

All lifespan, paralysis and stress resistance assays were initiated using 120 worms per group. This number is larger than the standard in the field (mostly 100 worms per group are used in such assays). Each experiment was repeated independently at least 3 times as detailed in the supplemental tables. For biochemical assays at least 3000 worms per sample were used.

Replicates

- You should report how often each experiment was performed
- You should include a definition of biological versus technical replication
- The data obtained should be provided and sufficient information should be provided to indicate the number of independent biological and/or technical replicates
- If you encountered any outliers, you should describe how these were handled
- Criteria for exclusion/inclusion of data should be clearly stated
- High-throughput sequence data should be uploaded before submission, with a private link for reviewers provided (these are available from both GEO and ArrayExpress)

Please outline where this information can be found within the submission (e.g., sections or figure legends), or explain why this information doesn't apply to your submission:

Each functional assay (lifespan, proteotoxicity and stress resistance) was independently repeated at least three times, by more than one person. All the information is provided at the supplemental tables. The number of repeats appear in figure that summarize more than one biological repeat (in some cases a representative experiment is displayed). No experiment was excluded.
Mass spectrometry data of differentially SUMOylated proteins was deposited at Pride.
To access the data:
Project Name: The Insulin/IGF Signaling Cascade Modulates SUMOylation to Regulate Aging and Proteostasis in C. elegans.
Project accession: PXD010011
Project DOI: Not applicable
Reviewer account details:

Username: reviewer98751@ebi.ac.uk

Password: kdRegLfk

Statistical reporting

- Statistical analysis methods should be described and justified
- Raw data should be presented in figures whenever informative to do so (typically when N per group is less than 10)
- For each experiment, you should identify the statistical tests used, exact values of N, definitions of center, methods of multiple test correction, and dispersion and precision measures (e.g., mean, median, SD, SEM, confidence intervals; and, for the major substantive results, a measure of effect size (e.g., Pearson's r, Cohen's d)
- Report exact p-values wherever possible alongside the summary statistics and 95% confidence intervals. These should be reported for all key questions and not only when the p-value is less than 0.05.

Please outline where this information can be found within the submission (e.g., sections or figure legends), or explain why this information doesn't apply to your submission:

Statistical analysis section appears at the end of Materials and Methods. The information appears in the text next to each experiment and the number of repeats (N) is displayed in the figures when appropriate. Accurate p values for all relevant experiments appear in the supplemental tables.

(For large datasets, or papers with a very large number of statistical tests, you may upload a single table file with tests, Ns, etc., with reference to sections in the manuscript.)

Group allocation

- Indicate how samples were allocated into experimental groups (in the case of clinical studies, please specify allocation to treatment method); if randomization was used, please also state if restricted randomization was applied
- Indicate if masking was used during group allocation, data collection and/or data analysis

Please outline where this information can be found within the submission (e.g., sections or figure legends), or explain why this information doesn't apply to your submission:

For all RNAi-based experiments worm eggs that were extracted from the same F_0 population were randomly placed on plates seeded with the indicated RNAi bacteria. When mutant worm strains were used (for instance EHC117 and EHC 118), worms that exhibit the roller selection marker were picked randomly. Each group was consisted of at least 120 worms. For qPCR experiment each group was of at least 1000 worms.

Additional data files ("source data")

- We encourage you to upload relevant additional data files, such as numerical data that are represented as a graph in a figure, or as a summary table
- Where provided, these should be in the most useful format, and they can be uploaded as "Source data" files linked to a main figure or table
- Include model definition files including the full list of parameters used
- Include code used for data analysis (e.g., R, MatLab)
- Avoid stating that data files are "available upon request"

Please indicate the figures or tables for which source data files have been provided:

A detailed experimental information appears in supplemental tables S1-S6. The relevant figure(s) appears at the title of each table.
